# Data-Driven Living Spaces’ Heating Dynamics Modeling in Smart Buildings using Machine Learning-Based Identification

**DOI:** 10.3390/s20041071

**Published:** 2020-02-16

**Authors:** Roozbeh Sadeghian Broujeny, Kurosh Madani, Abdennasser Chebira, Veronique Amarger, Laurent Hurtard

**Affiliations:** Université Paris-Est, LISSI Laboratory EA 3956, Senart-FB Institute of Technology, Campus de Senart, 36-37 Rue Charpak–F-77567 Lieusaint, France; madani@u-pec.fr (K.M.); chebira@u-pec.fr (A.C.); amarger@u-pec.fr (V.A.); hurtard@u-pec.fr (L.H.)

**Keywords:** system identification, smart building, artificial neural network, energy efficiency, black box modeling

## Abstract

Modeling and control of the heating feature of living spaces remain challenging tasks because of the intrinsic nonlinear nature of the involved processes as well as the strong nonlinearity of the entailed dynamic parameters in those processes. Although nowadays, adaptive heating controllers represent a crucial need for smart building energy management systems (SBEMS) as well as an appealing perspective for their effectiveness in optimizing energy efficiency, unfortunately, the leakage of models competent in handling the complexity of real living spaces’ heating processes means the control strategies implemented in most SBEMSs are still conventional. Within this context and by considering that the living space’s occupation rate (i.e., by users or residents) may affect the model and the issued heating control strategy of the concerned living space, we have investigated the design and implementation of a data-driven machine learning-based identification of the building’s living space dynamic heating conduct, taking into account the occupancy (by the residents) of the heated space. In fact, the proposed modeling strategy takes advantage, on the one hand, of the forecasting capacity of the time-series of the nonlinear autoregressive exogenous (NARX) model, and on the other hand, from the multi-layer perceptron’s (MLP) learning and generalization skills. The proposed approach has been implemented and applied for modeling the dynamic heating conduct of a real five-floor building’s living spaces located at Senart Campus of University Paris-Est Créteil (UPEC), taking into account their occupancy (by users of this public building). The obtained results assessing the accuracy and addictiveness of the investigated hybrid machine learning-based approach are reported and discussed.

## 1. Introduction and Related Works

In the context of the perspicacious decrease of fossil fuel resources and ongoing increase of energy consumption innate to the intensification of human urban activities during the last decades, the management of energy consumption in commercial and residential buildings has become a vital question. Regarding the works of [1] and [2], in the USA, the contribution of energy consumption in space heating was responsible for 43 percent in residential buildings in 2015, and in commercial buildings, this contribution was about 25 percent in 2012. This shows the huge slice of energy consumption related to space heating in the above-mentioned two sections. The recent enhancement in smart building energy management systems (SBEMSs) or smart building management systems (SBMS) by controlling and reducing the above-mentioned share of energy consumption is becoming the most efficient trend for facing energy consumption growth in residential and commercial buildings.

Smart building energy management systems (SBEMSs) in smart dwellings provide the inhabitants with advanced monitoring and control of the building’s functions and a clever way to manage heavy power-consuming appliances (as heating devices) in order to achieve energy efficiency while optimizing and preserving the inhabitants’ (or users’) comfortable environment [3].

Although the sensors’ quality and the technological features of the remote devices forming the physical part of the automated or smart buildings play an undeniable role in the performance of SBEMSs in optimizing the building’s energy consumption, the primary inefficiency of such systems in declining energy consumption is related to the quality of the models that bear either the identification of the relationship between the building’s behavior and the controller that hatches up the actions of implemented sensors and remote devices or to the excellence of the control strategy in charge of the building’s behavior control. Thus, the identification and modeling of the building’s operational dynamics remain key points in BMSs and especially in SBEMSs. On the other hand, the diversity of the involved factors (parameters) as well as their highly nonlinear variation make the identification and modeling of the dynamic behavior of a building a challenging task. Within this context and by considering that besides the living space’s intrinsic structural features, the occupation of the living space (by users or residents) may affect the model of heating dynamics of the concerned living space, we have investigated the design, implementation, and validation of a data-driven machine learning-based identifier supplied by the time-series prediction paradigm’s formalism. In fact, the human body continuously produces thermal energy, mostly in the form of heat radiation emission. Regarding black body law, a human in a sitting position and at about 1.80 m in height can emit 100 watts [4,5,6].

A number of works address model-free approaches coping with buildings’ heating. Related to conventional controllers, the authors of [7] introduced a control heating system for supporting the heating comfort of the user based on a very simple thermostatic controller (operating on an “on/off” strategy) with the help of a microcontroller. When the temperature is higher than the desired temperature, the fan will turn on, and when the temperature is lower than the desired temperature, the heater will turn on. The proposed simplistic control of the space heating operates on the difference between the desired temperature and actual temperature, and could be seen as a model-free heating approach. While taking advantage of its independency from the effective complexity of the concerned edifice’s hitting-dynamics, the proposed strategy is applicable to very specific homogenious living spaces, and cannot be generalized to more sophisticated buildings including heterogeneous living-spaces. In the work of [8], the investigator presents a gray-box methodology for thermal modelling of buildings. Gray-box modelling is a hybrid of data-driven and physics-based models, where coefficients of the equations from physics-based models are ajusted using data. The authors claim that the proposed methodology allows to capture the dynamics of the buildings while avoiding the effective complexity of the physics-based modelling, and results in simpler models. In fact, after first developing the individual components of the building such as temperature evolution, flow controller, and so on, the authors integrate these individual models into what they call the “complete gray-box model” of the building. The model has been validated using data collected from one of the buildings at Luleå, a city on the coast of northern Sweden. While using a simpler and generic model (compared with the physics-based complex heating models), the proposed approach remains far from convincing concerning its generalization to the other buildings.

The investigators of [9] propose a model-free and sensor-free *heating, ventilation and air-conditioning* (HVAC) control algorithm that uses simple user input (hot/cold) and adapts to changing office occupancy or ambient temperature in real time. As an alternative, the proposed strategy includes users in the HVAC control loop through distributed smart-phone based votes about their thermal comfort for aggregated control of HVAC. The developed iterative data fusion algorithm finds the optimal temperature in offices with multiple users and addresse techniques that can aggressively save energy by drifting indoor temperatures towards the outdoor temperature. The evaluation has been based on empirical data collected in 12 offices over a three-week period and showed that the proposed control may save up to 60% of energy at a relatively small increase in average occupant discomfort of 0.3 °C. While the idea is appealing, the concerned technique here also is very specific.

The control systems designed in [7,8,9] operate without any pre-knowledge of the living spaces that they are supposed to heat. In other words, the proposed solutions are based exclusively on data provided by temperature sensors within the frame of specific edifices for which the model of heating-dynamics is available. This makes the proposed models and issued controllers specific to the considered case studies, and thus not applicable to other structures (i.e., other buildings).

On the basis of the above-mentioned points, in the present article, we focus on the design and implementation of a data-driven machine learning-based identification of the building’s living-space dynamic heating conduct, taking into account the occupancy (by the residents) of the heated space. This step is necessary for pulling off a comprehensive (i.e., interpretable) model handling the dynamic heating conduct of a living space with and without human presence. The proposed data-driven machine learning-based identifier will be applied for modeling the dynamic heating conduct of a real five-floor building’s living spaces located at Senart Campus of University Paris-Est Créteil, taking into account their occupancy (by users of this public building).

From a general standpoint, identification approaches are divided into two main categories: white-box modeling (WBS) and black-box modeling (BBS) [10]. In WBS-based methods, the modeling of a system is performed on the basis of the formal relationship of the physical properties of the concerned system. If the main advantage of WBS-based methods remains their comprehensive and interpretable nature, however, often the effective complexity of real-world conditions causes WBS to lead to insolvable equations, and hence frequently to a strongly simplified issued model, making it quite far from the realistic behavior of the target system. In BBS-based methods, the modeling is done by mapping of an approximate behavior of the target system through the input–output relationship of that system. In contrast to WBS, if BBS-based methods achieve more accurate approximation of the effective complexity of the modeled system’s behavior, often they lead to a shortfall of comprehensive and interpretable foundation related to the issued model.

Numerous research works have been accomplished in the past decades within the areas of identification and modeling of nonlinear systems related to our purpose. Wiener and Hammerstein-type models [11], Volterra series [12], and machine-learning based approaches such as fuzzy logic-based models [13] and artificial neural network-based approaches [14] have been presented. The authors of [15] identify a solar heating system utilizing BBS based on what they call the “recursive prediction error method” (RPEM). It is on the basis of a state-space model. The target system (namely a solar heater) includes two inputs (solar radiation energy and speed of the fan) and one output (air’s temperature). They claim that the small amount of data necessary for the proposed approach is an advantage. However, the related simplicity of the target system and complicated expected behavior identification do not persuade the extendibility of the proposed approach to a realistic system including a large number of parameters (inputs and outputs).

In the work of [16], the identification of a heating system is done by investigation by means of an auto-regressive (ARX) model, auto-regressive and moving average (ARMAX) model, and Box–Jenkins (BJ) model. The target system includes a lamp and a metallic plate. It contains just one input (the lamp’s voltage) and one output (the metallic plate’s temperature). For the aforesaid case study, the authors used the system identification toolbox of MATLAB. However, the relative simplicity of the target system does not allow assessing the effectuality of the considered approach. It just presents that MATLAB’s system identification toolbox is able to imitate this uncomplicated case study example. Similarly, the authors of [17] used MATLAB’s identification toolbox for identification of the behavior of a boiler and heat exchanger transfer function. Nevertheless, the stated result does not end up with the accuracy of the target system identification. It results in a tough target device modeling. The authors of [18] provide the consequences of a dwelling’s thermal model identification. It includes two bedrooms heated by electrical baseboard heaters. Owing to the modeling of the target system, the authors used EnergyPlus (software for simulating the building energy system providing functional modeling of energy consumption for heating, cooling, ventilation, and lighting in buildings). The control signal was simulated by MATLAB. The Building Controls Virtual Test Bed open-source software (of Berkley Lab. [19]) is a free, available co-simulation software linking different simulation programs as EnergyPlus, Modelica (an object-oriented language for complex systems’ simulation [20]), and MATLAB/Simulink. In the account of the approximating dynamic of the system in Energy Plus, a low order state-space model is utilized. Concerning the identification of the system, they used N4SID subspace identification [21]. The authors in this investigation end up with a satisfactory average root-mean-square-error (RMSE) throughout ten reported simulated apartments. Nonetheless, they concluded that the time-consuming implementation makes it difficult to extend the proposed approach to more complicated systems.

The aforesaid investigations put emphasis on the pertinence of identification approaches for the modeling of buildings’ heating dynamics. Indeed, all of the referenced investigations underline the tough limitations of the overviewed solutions in matching the complex behavior of space heating systems in buildings. The main shortages are either related to the eager simplification of the actual operative complexity of involved equations, in order to ease their computational solutions, or inherent to the nonlinearity and outsized number of the involved parameters. If the analysis of the aforementioned research works highlights the diversity of the covered fields and applications, they confirm what we mentioned before related to the advantages and shortages of each category (i.e., WBS-like and BBS-like) of identification-based nonlinear systems’ modeling approaches. Meanwhile, the overviewed research works reveal the appealing capacity of the nonlinear autoregressive exogenous (NARX) model in modeling and forecasting complex systems’ behaviors. In fact, the proposed modeling strategy takes advantage, on the one hand, from the forecasting capacity of the time-series of the NARX model, and on the other hand, from the multi-layer perceptron’s (MLP) learning and generalization skills. If the NARX model has already been used for modeling in various paradigms, the originality of its application in the present article concerns its usage, and especially its closed-loop version, in the uninterrupted (i.e., continual) identification of the heating dynamics within a fully data-driven context. However, the additional novelties of the reported investigations, on the one hand, relate to the application of the aforementioned model for solving real-world problems addressing complex behaviors, and on the other hand, concern the effective implementation of the developed system by the use of standard technology (i.e., market available), overcoming complex technological obstacles.

Section 2 of this article presents the method and concepts of the proposed data-driven identification approach. Section 3 details the implementation of the issued method on SBEMS of the above-mentioned five-floor experimental building. The experimental setup, the experimental protocol, and the obtained results are presented and discussed. Finally, Section 4 concludes the article.

## 2. Machine Learning-Based Identification of the Heating Dynamics of the Living Space

Before bestowing the proposed living space heating dynamics identification approach, we consider the following work hypothesis relating to the identification strategy:

- The concerned living space is supposed to be part of a typical building including various quarters (such as flats and rooms for a residential building or working spaces, office rooms, classrooms, and practice rooms for a public building, and so on).
- The building is supposed to be heated by a central heater supplying radiators located in the aforementioned living spaces.
- The regulation is supposed to be done by a conventional controller adjusting the radiators’ valves versus the magnitude of the outdoor temperature and the target (i.e., desired) indoor temperature.
- The target model considers the system to be identified as an overall system including the heat transmitters (radiators) and the heating space.
The concerned living space is supposed to contain an amount of N (with $0\leq N\leq N_{Max}$) occupants (residents or users). N=0 corresponds to an empty living space, while $N=N_{Max}$ characterizes a fully occupied living space. Thus, $N_{Max}$ corresponds to maximum capacity of the living space and is determined according to the construction norms and occupation regulations.

Thus, within the aforementioned work hypothesis, the considered parameters are as follows: “Valve-position at time t” (denoted by $\vartheta_{P}\left(t\right)$), providing the heated water’s flow (expressed as a normalized ratio of debit versus the maximum debit of the valve); “Outdoor-Temperature measured at time t” (denoted by $T_{Out}\left(t\right)$, expressed in °C); “Indoor-Temperature measured at time t” (denoted by $T_{In}\left(t\right)$, expressed in °C); and “Occupancy-Rate at time t” (denoted by $O_{CC}\left(t\right)$, expressed in %).

**Figure 1:**
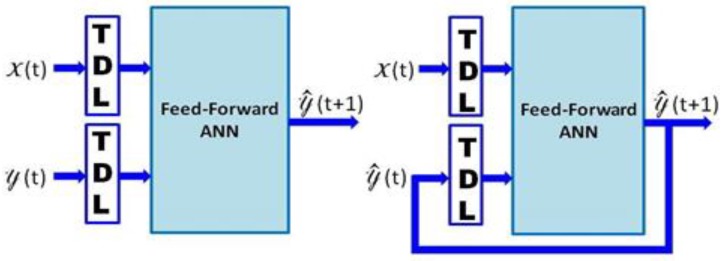
General artificial neural network (ANN)-based nonlinear autoregressive exogenous (NARX) model: open-loop (left) and closed-loop (right) architectures.

As mentioned in the introductory section, the identification method of the proposed system is accomplished by an MLP-based NARX ([22,23,24]) with a feed-forward back-propagation learning algorithm ([25,26]). Equation (1) and (2) respectively specify the overall open-loop NARX model, where F(·) is the activation function of the ANN, $\hat{y}\left(t+1\right)$ is the estimated (i.e., predicted) output, y(t) is the actual output value of the model (i.e., at time t), y (t−1),⋯,y (t−n) are n-past values of the y(t), x(t) is the present input value, and y(t),y (t−1),⋯,y (t−m) are the actual and tapped delayed exogenous inputs in m-past input values. Figure 1 illustrates the overall schema of the NARX model ${\hat{T}}_{In}\left(t\right)$.

(1) $$\hat{y}\left(t+1\right)=F\left(y\;\left(t\right),\;y\;\left(t-1\right),\cdots ,y\;\left(t-n\right),\;x\;\left(t\right),\;x\;\left(t-1\right),\;\cdots ,\;x\;\left(t-m\right)\right)$$

(2) $$\hat{y}\left(t+1\right)=F\left(\hat{y}\left(t\right),\;\hat{y}\left(t-1\right),\cdots ,\hat{y}\left(t-n\right),\;x\;\left(t\right),\;x\;\left(t-1\right),\;\cdots ,\;x\;\left(t-m\right)\right)$$

**Figure 2:**
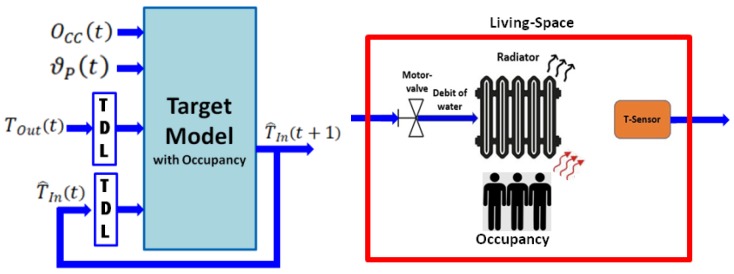
Proposed identification architecture of the target heating model (left-side) and general diagram of the modeled living space including the influence of its occupancy (right-side).

Figure 2 reveals the proposed identification structure of the target heating model, taking into account the above-stated work hypothesis including the influence of the occupancy. The learning process is performed by utilizing the open-loop NARX scheme. The learning dataset contains the operation of the real system’s sequences within different valve positions (i.e., $\vartheta_{P}\left(t\right)$), providing various heating powers, the occupancy-rate at time t (i.e., $O_{CC}\left(t\right)$), the actual and m-past measures of outdoor temperature (i.e., $T_{Out}\left(t\right)$, $T_{Out}\left(t-1\right)$, … and $T_{Out}\left(t-m\right)$), and the actual and n-past values of indoor temperature (i.e., $T_{In}\left(t\right)$, $T_{In}\left(t-1\right)$, … and $T_{In}\left(t-n\right)$).

The influence of occupancy is modeled through the following hypothesis: *occupancy of the living space by residents increases the effective overall heating power*. The right-side picture of Figure 2 gives the general diagram of the modeled living-space within the aforementioned hypothesis. In other words, we assume that occupation of the living space by residents (i.e., bodies providing additional heating sources) is equivalent to an incensement of the heating device’s nominal power. To determine the above-mentioned equivalent nominal power, we exploit the usual policy of setting the adequate heater’s nominal power versus the living space’s characteristics. In fact, building designers determine the adequate heater’s nominal power on the basis of the volume of the concerned living space (i.e., the living space that is supposed to be heated by the heating device) by keeping constant a parameter called “*Heating Ratio*” (denoted by $HR_{0}$, expressed in W/m^3^), defined by Equation (3), where $P_{No}$ denotes the heating device’s nominal power (which depends on the technological and structural features of the heating device) and $V_{LS}$ denotes the volume of the living space (room, and so on). The appropriate value of $HR_{0}$ is determined versus construction norms (materials, processes, and so on used for constructing the concerned building) and urban, social, and environmental regulations (imposed by legal authorities).

(3) $$HR_{0}=\frac{P_{No}}{V_{LS}}$$

Taking into account the hypothesis related to the occupancy’s influence, Equation (3) may be extended in terms of Equation (4) taking into account the occupancy’s influence. In this equation, HR(N) denotes the “*Heating Ratio*” taking into account the occupancy, $P_{OCC}\left(N\right)$ states for additional heating power provided by the living space’s occupancy (with $P_{OCC}\left(N=0\right)=0$), and $V_{LS}$ denotes the volume of the living space (room, and so on).

(4) $$HR\left(N\right)=HR_{0}+\frac{P_{OCC}\left(N\right)}{V_{LS}}$$

One can notice that $HR\left(N=0\right)=HR_{0}$ corresponds to the *Heating Ratio* of the same living space when it is empty. We determine $P_{OCC}\left(N\right)$ through a fuzzy-logic-based concept by considering, on the one hand, three categories (fuzzy intervals) of living spaces (i.e., three fuzzy intervals of living space’s volume), and on the other hand, by considering five categories of occupancy-rate (denoted by $O_{CC}$, expressed in %). Namely, the three categories for living space’s volume are as follows: “Large”, “Medium”, and “Small”. The five categories of occupancy-rate are as follows: “Empty” (corresponding to $O_{CC}=0\%$), “Small occupation” (i.e., $O_{CC}=25\%$), “Medium occupation” (i.e., $O_{CC}=50\%$), “High occupation” (i.e., $O_{CC}=75\%$), and “Full” (i.e., $O_{CC}=100\%$). Within this formulation, Equation (4) may be written in terms of Equation (5), where $V_{LS}^{F}$ denotes the “*fuzzy value*” of $V_{LS}$.

(5) $$HR\left(O_{CC}\right)=HR_{0}+\frac{P_{OCC}\left(O_{CC}\right)}{V_{LS}^{F}}$$

The main advantage of such a formulation is that the above-mentioned intervals may be quantified by the use of data-driven statistical clustering methods, reflecting the reality of the concerned building’s usage (occupancy). Thus, the equivalent nominal power of a given living space occupied by N bodies may be estimated as $HR\left(O_{CC}\right)\times V_{LS}$.

Flooding back to the hypothesis we made related to the effect of the living space’s occupancy, another way of interpreting the aforementioned hypothesis is to note that the occupancy of the living space by residents will decrease the required time for reaching the target indoor temperature. In other words, the higher the living space’s occupancy, the shorter the necessary delay to heat it. In order to quantify this, we introduce what we call “*Heating Slop*” (denoted by h(t) and expressed in °C/s), defined as the derivative of $T_{In}\left(t\right)$ versus the time and approximated by Equation (6) within the context of a discrete sampling (i.e., discrete measurement) of the indoor temperature. In Equation (6), $T_{In}\left(t_{k}\right)$ and $T_{In}\left(t_{k-1}\right)$ stand for consecutive values of indoor temperature (supposed to be provided by the temperature sensor at times $t_{n}$ and $t_{n-1}$, respectively) and $\Delta t=t_{k}-t_{k-1}$.

(6) $$h\left(t\right)=\frac{T_{In}\left(t_{k}\right)-T_{In}\left(t_{k-1}\right)}{\Delta t}$$

It is pertinent to notice that an escalation of the living space’s occupancy (introducing additional bodies and thus additional sources of heating) or decrease of occupancy will result in the so-called heating slop’s modification: the higher the living space’s occupancy, the stronger the heating slop. Within the general standpoint, and as formulated by Equation (6), h(t) is time-dependent, and thus may vary along with time. However, because of the fact that the heating of buildings abides by slow dynamics, often h(t) remains constant (with regard to the time), albeit its value would vary along with the valve-position (i.e., with $\vartheta_{P}\left(t\right)$) that controls the heating device’s actual power. On the basis of the aforementioned points, actually, the predicted indoor temperature (i.e., ${\hat{T}}_{In}\left(t_{k+1}\right)$) may be computed from Equation (7), where $\hat{h}\left(t\right)=f\left(\vartheta_{P}\left(t\right)\;,\;O_{CC}\right)$ denotes the identified (estimated) value of h(t).

(7) $${\hat{T}}_{In}\left(t_{k+1}\right)=\hat{h}\left(t\right)\;\left(t_{k+1}-t_{k}\right)$$

## 3. Implementation of the Proposed Living-Spaces’ Dynamic Heating Model

As has been mentioned, a real five-floor building located at Senart Campus of University Paris-Est Créteil (UPEC) served as an experimental platform for the evaluation and validation of the proposed model. The concerned building (namely Building A of the campus) is a fully automated building hosting the Electrical Engineering and Industrial Informatics Department of Senart-Fontainebleau Institute of Technology of UPEC. The building (i.e., system to be identified) is heated by a conventional central heater supplying radiators (i.e., heating devices) located in various living spaces (namely, office rooms, classrooms, practical rooms, and so on) of the building. The central heater is common to three buildings of the campus, and thus the control of the local heating devices of the concerned buildings (including Building A) is performed through the local valves of each radiator. The two other buildings are conventional buildings (i.e., not automated) and the sole Building A is automated. In fact, Building A is equipped with numerous sensors and connected devices allowing the recording of data related to environmental information (such as temperatures in each living space and the outdoor temperature) and the operational states of whole installed connected devices (such as radiators’ valves). Four different kinds of sensors outfit each living space (including corridors) the entire five floors of this building: “temperature sensors” (TSs), “magnetic sensors” (MSs), “presence detectors” (PDs), and “luminance sensors” (LSs). The main connected devices (actuator) deployed in the aforementioned experimental building are as follows: “motor valves” (MVs), which control radiators supplied by the abovementioned central water-flowed heating system, and connected “lighting elements” (LEs).

**Figure 3:**
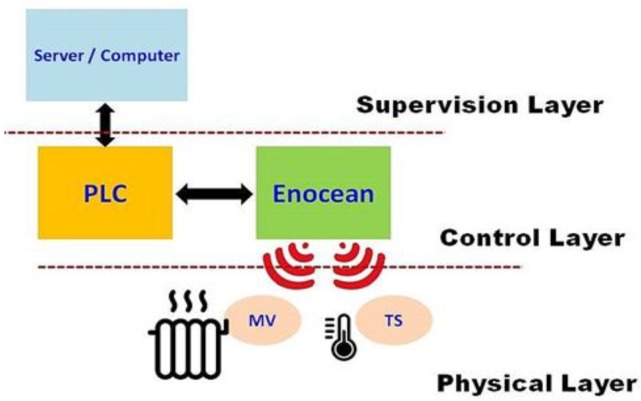
Implementation diagram of the heating system equipping Building A of Senart Campus of University Paris-Est Créteil (UPEC). PLC, programmable logic controller; MV, motor valve; TS, temperature sensor.

Sensors and connected devices concerned by the purpose of the present paper are TSs and MVs. They use “EnOcean” technology; an energy harvesting wireless technology provided by EnOcean [27]. EnOcean-technology-based modules fuse micro-energy converters with ultralow power electronics and reliable wireless communications, allowing to provide self-powered wireless sensors or actuators for building energy management systems as well as for industrial applications. Figure 3 presents the implementation diagram of the concerned building (Building A) heating system.

The connected heating system includes three operational layers:

- Supervision layer (SL): It consists of a PC including TopKapi server supervision software (a supervisory control and data acquisition software), which acts as a supervision agent. It also includes a number of adequate interface agents (software units) concerning the control layer and storage memory [28]. It is relevant to note that, while nowadays micro-controllers are able to handle diverse computational skills, they may still be limited regarding computational needs relating to the context of the presented work. In fact, in our work, we deal with machine learning-based identification, where a number of computational tasks need improved computational ability (especially for the training task). Actually, the effective adaptability to the real-usage context of the system would require updating the models’ parameters versus the evolution of effective conditions (i.e., bring up to date the system’s “knowledge”). That is why the choice was directed toward integrating a server. Moreover, the target system addresses smart-buildings’ context, and thus would deal with a rather large number of living spaces. This reinforces the choice of superior computational ability.
- Control layer (CL): This layer contains the programmable logic controller (PLC) and EnOcean modules (pilots and interfaces) necessary to conduct the related sensors and devices composing the physical layer [29]. The concerned PLC is a “WAGO-I/O-SYSTEM” belonging to the family of ETHERNET programmable Fieldbus controllers distributed by WAGO company [30]. It supports both MODBUS/TCP and a wide variety of standards ETHERNET/IP protocols in order to integrate easily into various IT environments.
- Physical layer (PL): It consists of the aforesaid sensors and actuators devices.

Composing the heating control chain of the SBEMS of the aforementioned fully automated experimental building, the CL and PL are replicated for each floor, making possible the set up collecting data characterizing the heating state of each living space of the building and controlling valve position of each heating device (radiator) in the building through the five PLCs (one for each floor). The proposed identification approach was implemented in the SBEMS of the aforementioned fully automated experimental building.

For evaluation of the proposed identification strategy and the issued model, two experimental assessments were considered. The first one appraises the obtained model’s “one-step prediction” (OSP) accuracy and the second one sizes up the ability of the issued model on “multi-step prediction” (MSP). The purpose of OSP aims to predict the living space’s immediate upcoming indoor temperature from its previous history. Therefore, open-loop as well as closed-loop architectures could be used. Meanwhile, the objective in MSP relates to the prediction of several successive future steps of the concerned living space’s indoor temperatures, and thus the open-loop architecture remains no more pertinent.

## 4. Experimentation and Results

### 4.1. Experimental Protocol’s Description

**Figure 4:**
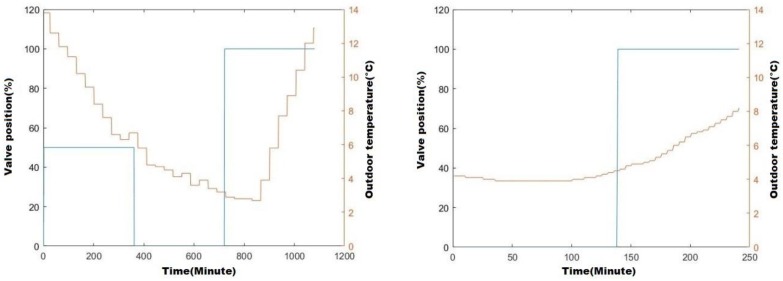
Experimental data collection conditions comply with the empty working space (left-side diagram) and fully occupied working space (right-side diagram), respectively, showing the valve position and outdoor temperature–time history.

Both of the two aforementioned evaluations are performed in keeping with the same experimental protocol. This protocol considers a living space of Building A belonging to the category of “middle-size” working spaces of this building (i.e., $V_{LS}^{F}=“Medium”$) able to soak up 28 residents (individuals). The considered living space is equipped with a 3 kW heating device (namely a 3160 W radiator supplied by the central heater), responding to the construction and legal norms applicable to this category of working spaces. The collected data are remote values (time history) of outdoor temperature $T_{Out}\left(t\right)$, radiator’s valve position $\vartheta_{P}\left(t\right)$, and indoor temperature $T_{In}\left(t\right)$. Two sets of experimental data were collected. The data sampling period is one minute, meaning that the value of each considered parameter is collected periodically every 60 s. Figure 4 depicts the experimental conditions showing the valve’s position and living space’s temperature evolution, respectively.

The first one, which we tag as “Empty-working-space” (EWS), includes the time history of the aforementioned parameters’ values during 18 h (i.e., 1080 min) when the considered working space is empty (i.e., $O_{CC}\left(t\right)=0\%$). The left-side diagram of Figure 4 depicts the experimental conditions, showing the local heating device’s operational conduct and the outdoor temperature’s evolution during the data collection sequence. As is visible from this diagram, the working space was heated over six hours by the radiator operating with 50% of its nominal power (i.e., 

$\vartheta_{P}\left(t\right)=50\%$ during the first six hours). Then, the heating was stopped during the six next hours (i.e., $\vartheta_{P}\left(t\right)=0\%$ from $t=360{}^{\prime }$ to $t=720{}^{\prime }$). Finally, during the last period of six hours, the work space was heated by the radiator developing its maximum nominal power (i.e., $\vartheta_{P}\left(t\right)=100\%$ from $t=720{}^{\prime }$ to $t=1080{}^{\prime }$).

The second one, which we tag as “Fully-occupied-working-space” (FWS), includes the time history of the aforementioned parameters’ values when the same working space is occupied by 28 individuals (i.e., $O_{CC}\left(t\right)=100\%$) during 100 min of a period of four hours. The right-side diagram of Figure 4 depicts the experimental conditions related to this second set of collected data, showing the local heating device’s operational conduct and the outdoor temperature’s evolution during the data collection sequence. As is visible from this diagram, the working space is not heated during the first 140 min, assuming that the working space is empty and thus does not need to be heated. Then, while being fully occupied (i.e., $O_{CC}\left(t\right)=100\%$) during next period of 100 min, it is heated by the radiator developing its maximum nominal power (i.e., $\vartheta_{P}\left(t\right)=100\%$ from $t=140{}^{\prime }$ to $t=240{}^{\prime }$).

A part of the collected datasets serves for training of the proposed ANN-based NARX identifier and a ratio of the collected data is used as testing data. The parameters of ANN for all constructed models are as follows:

- Identification of empty working space was performed using ANN including one hidden layer with a size of 5 (number of neurons in the hidden layer). The number of neurons in the hidden layer was set empirically. Related to the training and validation, 85% of data was utilized for training and 15% for testing.
- Identification of fully occupied working space was performed using ANN including one hidden layer with a size of 10 (number of neurons in the hidden layer). The number of neurons in the hidden layer was set empirically. Related to the training and validation, 90% of data was utilized for training and 10% for testing.

The training and testing operations were repeated 10 times for each collected dataset. The evaluation of the obtained model’s accuracy was done based on mean squared error (MSE) and mean absolute error (MAE) criteria. It is illustrated by Equations (8) and (9), respectively, where N is number of samples, *y_i_* is the effectively recorded data, and *ŷ_i_* represents the estimated (predicted) value.

(8) $$MSE=\frac{1}{N}\overset{N}{\underset{i=1}{\sum }}{\left(y_{i}-{\hat{y}}_{i}\right)}^{2}$$

(9) $$MAE=\frac{1}{N}\overset{N}{\underset{i=1}{\sum }}\left|y_{i}-{\hat{y}}_{i}\right|$$

It is relatable to note that the first trial concerned the experimental settlement of the number of time-delayed data samples to be considered in the prediction task. The concerned data correspond to the three-hour sequence of the EWS when the considered working space is heated by the radiator developing its nominal power, leading to a swell of 2.7 °C of the indoor temperature (i.e., data recorded between $t=720{}^{\prime }$ and $t=900{}^{\prime }$). A part of extracted sequence was used for identification and the rest of the sequence’s data served for testing the forecasting ability of the issued model. The amount of data related to the machine learning task links the representativeness of the collected data regarding the considered environmental and human (i.e., dwellers) factors involved in the constructed models (i.e., influencing the system’s conduct). Typical delays of living spaces’ heating (i.e., temperature variation) reflecting a representative sequence of the buildings operational behavior are between two hours (for a fully occupied living space) and six hours (for an empty living space). Taking into account the implementation technologies and the precision of the deployed sensors (i.e., 0.1 °C for temperature sensors and 1% for radiators’ motor valves), the sampling period (data acquisition every minute), and the involved building’s heating dynamics, this leads to to a sufficient amount of data representative of the system’s conduct. Besides the above-mentioned, the proposed system’s implementation architecture allows a versatile collection of complementary data at any time or in a continuous way.

**Figure 5:**
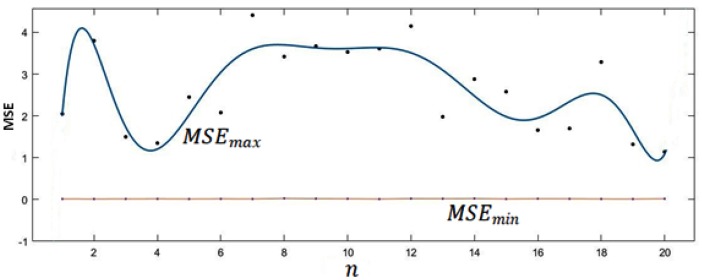
Minimum and maximum mean squared error (MSE) ($MSE_{min}$ and $MSE_{max}$) versus the number of considered time-delayed data samples involved in the prediction task (n).

Twenty models were constructed, differing with regard to the number of the considered time-delayed data samples involved in the prediction task. Figure 5 depicts the obtained results representing the minimum and maximum value of MSE versus the number of the considered time-delayed data samples involved in the prediction task. The above-stated number of models has been aspired by technical features of the deployed implementation technology’s features. Actually, the sampled data are transmitted by the deployed module every 18 min with the already mentioned sampling period of 60 s. By taking this fact into account, we aimed to study the plausible influence on the forecasting accuracy of considered time-delayed data (from 1 to 20) related to the involved parameters (i.e., indoor temperature, valve position). On the other hand, each model has been trained and tested 10 times, allowing a quantitative (i.e., statistics) evaluation of the aforementioned possible influence.

If $MSE_{min}$ remains within the interval [0.12 , 0.26], the lowest obtained $MSE_{max}$ values are obtained with n=4 and n=20. Taking account of the implementation’s computational constraints, n=4 (corresponding to $MSE_{min}≅0.12$ and $MSE_{max}<1.5$) appears to offer a suitable compromise. According to the obtained results (shown in Figure 5), the experimental evaluation was performed using n = 4, stressing our choice toward a lower computational complexity.

### 4.2. Experimental Results

The two constructed datasets have served for evaluating OSP as well as MSP models of the considered working space’s heating conduct. Following the experimental protocol described in the previous section, the data-driven learning-based issued model was pointed out using data partly from EWS and partly from FWS. Two operational scenarios corroborating experimental recorded data conditions (i.e., those depicted by Figure 4) were considered:

1. The first case study, focusing OSP accuracy, considers two painless operational situations:
  a The first situation assumes that the considered working space is empty (i.e., $O_{CC}\left(t\right)=0\%$), the outdoor temperature is up to $T_{Out}\left(t\right)=10\;{}^{\circ}C$, and the indoor temperature at the beginning of the heating sequence is 20.86 °C (i.e., $T_{In}\left(t=0\right)=20.86\;{}^{\circ}C$). Starting under the above-mentioned conditions, the considered empty working space is supposed to be heated during two hours (i.e., during 120′) by the radiator developing its nominal heating power (i.e., $\vartheta_{P}\left(t\right)=100\%$).
  b The second situation assumes that the considered working space is fully occupied by 28 residents (i.e., $O_{CC}\left(t\right)=100\%$), the outdoor temperature is up to $T_{Out}\left(t\right)=5\;{}^{\circ}C$, and the indoor temperature at the beginning of the heating sequence is 21.80 °C (i.e., $T_{In}\left(t=0\right)=20.80\;{}^{\circ}C$). Starting under the above-mentioned conditions, the considered occupied working space is supposed to be heated during 350′ by the radiator developing its nominal heating power (i.e., $\vartheta_{P}\left(t\right)=100\%$).
2. The second case study focuses on MSP accuracy evaluation, considering two more tricky situations:
  c Assuming that the considered working space is empty (i.e., $O_{CC}\left(t\right)=0\%$), the first situation of this second case-study presumes that the working space is heated in accordance with the left-side diagram of Figure 4. In other words, it supposes that the radiator heating the considered living space heats it during six hours developing 50% of its nominal power (i.e., $\vartheta_{P}\left(t\right)=50\%$ for $t\;\in \left[0^{\prime },\;360{}^{\prime }\right]$), and then after a six-hour halt (i.e., $\vartheta_{P}\left(t\right)=0\%$ for $t\;\in \left[361{}^{\prime },\;720{}^{\prime }\right]$), it reheats this same working space during an additional six hours developing its whole nominal power (i.e., $\vartheta_{P}\left(t\right)=100\%$ for $t\;\in \left[721{}^{\prime },\;1080{}^{\prime }\right]$). The outdoor temperature (i.e., $T_{Out}\left(t\right)$) is supposed to vary during those 18 h within the interval [2 °C , 14 °C], also in line with the left-side diagram of Figure 4. The indoor temperature at the beginning of the heating sequence is 19.60 °C (i.e., $T_{In}\left(t=0\right)=19.60\;{}^{\circ}C$).
  d Presuming that the considered working space is empty at the beginning (i.e., $O_{CC}\left(t\right)=0\%$ at t = 0), the second situation of this second case-study assumes that the concerned working space becomes fully occupied during 100 min and is reheated in accordance with the right-side diagram of Figure 4. In other words, it supposes that the radiator is off during the first 140 min when the living space is empty (i.e., $\vartheta_{P}\left(t\right)=0\%$ and $O_{CC}\left(t\right)=0\%$ for $t\;\in \left[0{}^{\prime },\;140{}^{\prime }\right]$), and it heats the considered living space during 100 min when the room is fully occupied, developing its nominal power (i.e., $\vartheta_{P}\left(t\right)=100\%$ and $O_{CC}\left(t\right)=100\%$ for $t\;\in \left[141{}^{\prime },\;240{}^{\prime }\right]$). The outdoor temperature (i.e., $T_{Out}\left(t\right)$) is also supposed to vary within the interval [4 °C, 8 °C] in line with the right-side diagram of Figure 4. The indoor temperature at the beginning of the heating sequence is 17.25 °C (i.e., $T_{In}\left(t=0\right)=17.25\;{}^{\circ}C$).

**Figure 6:**
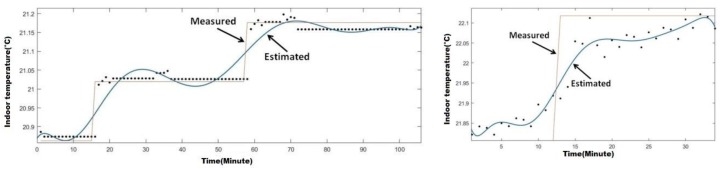
Model-based, one-step predicted indoor temperature time history when the living space is empty (left-side) and when it is fully occupied (right-side).

**Figure 7:**
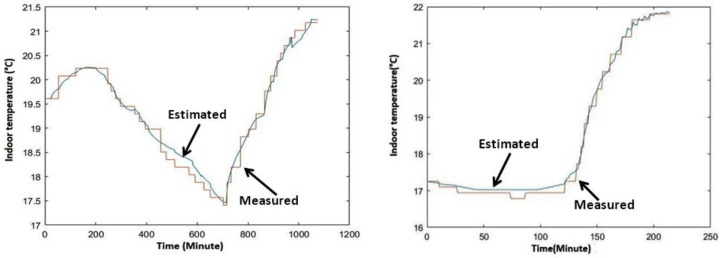
Model-based, multi-step predicted indoor temperature time history when the living space is empty (left-side) and when it is fully occupied (right-side).

Figure 6 and Figure 7 show the obtained results related to the two above-mentioned case-studies. Concerning the first case-study, the left-side diagram of Figure 6 plots the estimated (i.e., model-based OSP) indoor temperature and measured (i.e., real) indoor temperature of the considered living space when it is empty. The right-side diagram of this same figure gives model-based predicted and real indoor temperature’s values when the considered living space is fully occupied. Linking the second case-study, the left-side diagram of Figure 7 gives the estimated (i.e., model-based MSP) indoor temperature and measured (i.e., real) indoor temperature of the considered living space when it is empty. The right-side diagram of this same figure depicts the model-based, multi-step prediction of indoor temperature values and the measured temperature when the living space is fully occupied during 100 min.

Table 1 summarizes the overall accuracy of OSP and MSP models of the considered working space’s heating conduct. As expected, the OSP model of the aforementioned heated living space forecasts the upcoming value of the indoor temperature with less than 0.2 °C blunder compared with the measured value. Actually, the achieved high prediction accuracy is because of the fact that, in the OSP model, the prediction is performed using the four effectively-measured past values of the indoor temperature, and thus representing the effective time-history of indoor temperature’s evolution. However, anchored in an open-loop NARX scheme, the main shortage of this model would appear when a longer-term forecasting of indoor temperature is needed.

**Figure 8:**
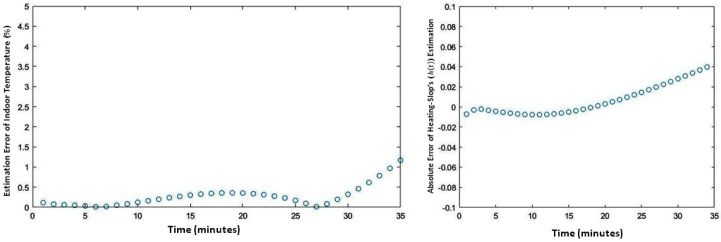
Indoor temperature estimation’s absolute error (left-side) and heating slop’s forecasting error (right-side).

The MSP model of the aforementioned heated living space forecasts the upcoming value of the indoor temperature with lower accuracy compared with the OSP model; an average error up to 0.4 °C, while attaining, for some long-term predicted values of indoor temperature, an error exceeding 1.23 °C. The finer analysis of indoor temperature’s forecasting, supported by the results depicted in Figure 8, provide the incentive of the observed gap. The left-side diagram of Figure 8 reports the estimation’s absolute error for 35 consecutive estimated indoor temperatures (i.e., prediction of the 35 upcoming values of indoor temperature) of the modeled heated working space. The right-side diagram of Figure 8 plots the forecasting error of the so-called “heating slop” (i.e., h(t)), defined and introduced in Section 2. In fact, as visible from those diagrams, if both estimation errors (i.e., the estimation error relative to $T_{In}\left(t\right)$ and the forecasting error related to h(t) values’ estimation) remain close to zero for short-term (5 values) and middle-term (15 values) forecasted indoor temperature values, both of them admit a continuously increasing evolution for long-term predicted values, especially those surpassing the next thirty-minute predicted period. Actually, within such a longer-term prediction requirement, the generalization of MLP neural net seems to reach its limitation regarding the learning dataset.

**Figure 9:**
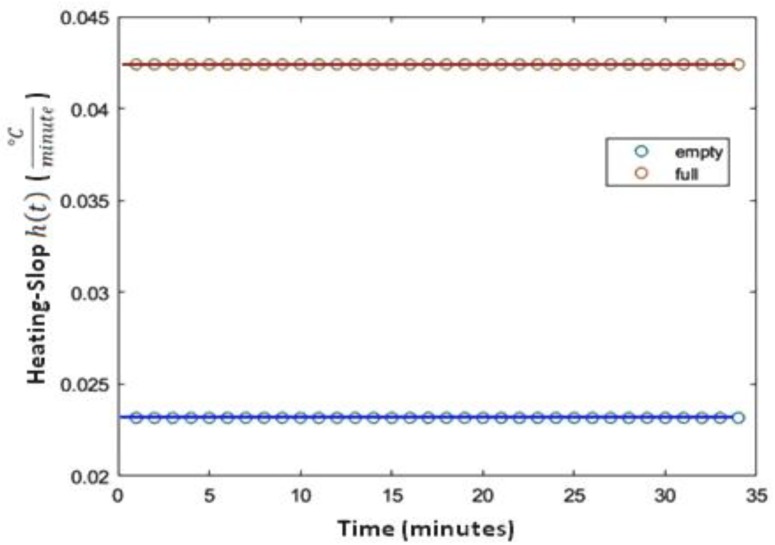
Heating slop when the living space is empty and when it is fully occupied.

Figure 9 depicts the heating slop (i.e., h(t)) when the considered living space is heated by its radiator in two different situations: the blue curve corresponds to h(t) when the living space is empty and the other curve relates to the situation when the same heated space is fully-occupied. It is pertinent to notice that the average estimated heating slop for the empty living space is $\bar{h\left(t\right)}=0.015\;\frac{{}^{\circ}C}{minute}$, while, for the same fully occupied living space, it is more than four times stronger ($\bar{h\left(t\right)}=0.042\;\frac{{}^{\circ}C}{minute}$ ), showing up the occupancy’s impact on the considered living space’s heating dynamics. In fact, the indoor temperature of the considered living space heated with a same radiator will increase up to 0.5 °C in 35 min when it is empty, while it will increase up to 1.5 °C when the living space is fully occupied.

**Figure 10:**
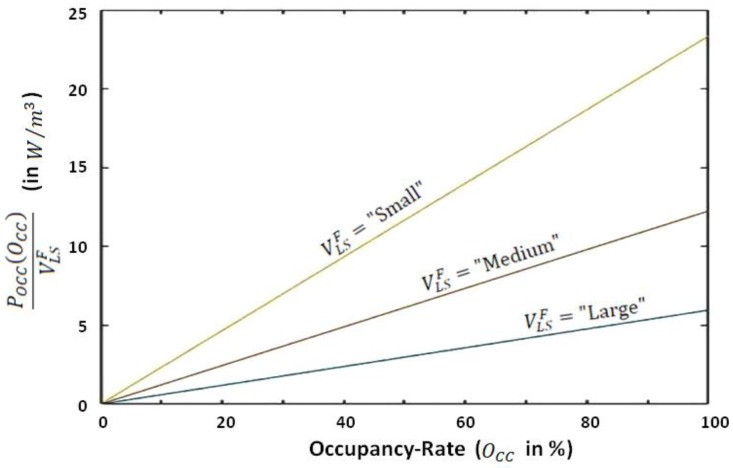
$\frac{P_{OCC}\left(O_{CC}\right)}{V_{LS}^{F}}$ (introduced in Equation (5) of the second section) versus the occupancy rate for the three considered categories of living spaces of Building A.

Finally, Figure 10 shows $\frac{P_{OCC}\left(O_{CC}\right)}{V_{LS}^{F}}$ (introduced in Equation (5) of the second section) versus the occupancy rate for the three considered categories of living spaces of Building A, where $O_{CC}=100\%$ corresponds to the occupancy of each considered living space category’s by up to 28 individuals. As an example, according to this diagram, 28 individuals occupying a medium living space of this building, equipped with a 3 kW radiator, would correspond to an additional heating power of 1170 W.

## 5. Conclusions

Considering that, alongside the living space’s intrinsic structural features, the occupancy of the living space (by users or residents) may affect the model of heating dynamics of the concerned living space, we have investigated the design, implementation, and validation of a data-driven machine learning-based identifier supplied by the time-series prediction paradigm’s formalism. The proposed data-driven machine learning-based identifier was applied for modeling the dynamic heating conduct of a real fully automated five-floor building’s living spaces located at Senart Campus of University Paris-Est Créteil, taking into account their occupancy (by users of this public building). The proposed modeling strategy takes advantage, on the one hand, of the time-series’ forecasting capacity of the NARX model, and on the other hand, of the multi-layer perceptron’s (MLP) learning and generalization skills.

If, as expected, the one-step-prediction (OSP) model, operating on the basis of an open-loop scheme, achieved high prediction accuracy in forecasting of the upcoming value of the indoor temperature (i.e., less than 0.2 °C blunder comparing to the measured value), anchored in an open-loop NARX scheme, its main shortage appears when a longer-term forecasting of indoor temperature is required; especially, when the target model is used for designing an adaptive heating control strategy. Achieving a lower accuracy compared with the OSP model (i.e., an average error up to 0.4 °C and a maximum error of 1.23 °C for long-term prediction), the multi-step-prediction (MSP) model, operating in closed-loop, represents an attractive compromise for longer-term forecasting of the dynamic heating behavior, and thus offers an appealing perspective for designing adaptive heating controllers for SBEMSs.

The achieved results stress several appealing issues related to the denotation of these results as well as the status of the NARX-based forecaster regarding data-driven identification of heating dynamics in real smart-buildings. The first points come across the ability of the proposed approach in the modeling complex thermal conduct of buildings, including the effect of inhabitants’ presence on the discrepancy of their heating dynamics. In fact, this is visible through the obtained MSE and MAE values, highlighting a prediction of indoor temperature with a less than 0.2 °C blunder compared with the measured value. This foretells the perspective of effectual usage of the proposed approach for designing data-driven adaptive controllers of buildings’ heating behavior versus the context of their usage by potential residents. The second remark relates to the possibility of a standard-technology-based effective implementation of this investigated machine learning-based identifier in authentic smart-buildings, taking advantage of the robustness of those standard (and market available) technologies and avoiding the complexity and cost of designing specific implementation policies. Finally, the last mention goes to the accuracy of the achieved predictions related to well short-term (i.e., one-step) as well as long-term (i.e., closed-loop) forecasters. Another attractive feature, arising from the theoretical foundation of the proposed approach, relates to the comprehensive interpretation of the living space’s occupancy effect, with a quantitative appreciation of its influence on the smart-building’s heating conduct’s deviation.

## Figures and Tables

**Table 1:** Estimation’s maximum and minimum mean squared error (MSE) and mean absolute error (MAE) with four delayed samples considered for each involved parameter (i.e., n=4). OSP, one-step prediction; MSP, multi-step prediction.

	Tests	OSP	MSP (Test Data)	MSP (Whole the Data)
Errors				
$MSE_{min}$		0.0006	0.021	0.020
$MAE_{min}$		0.01	0.12	0.11
$MSE_{max}$		0.03	2.45	0.25
$MAE_{max}$		0.15	1.23	0.44

## References

[1] U.S. EIA (2017). Energy Use in Commercial Buildings-Energy Explained, Your Guide To Understanding Energy—Energy Information Administration. https://www.eia.gov/energyexplained/index.php?page=us_energy_commercial.

[2] Space Heating and Water Heating (EIA). https://www.eia.gov/todayinenergy/detail.php?id=37433.

[3] Alhamoud A., Ruettiger F., Reinhardt A., Englert F., Burgstahler D., Böhnstedt D., Gottron C., Steinmetz R. An Intelligent System for Energy Saving in Smart Home. Proceedings of the 39th Annual IEEE Conference on Local Computer Networks Workshops.

[4] James D.H. (1934). The Radiation of Heat from the Human Body New York Hospital.

[5] Fang J.S., Hao Q., Brady D.J., Shankar M., Guenther B.D., Pitsianis N.P., Hsu K.Y. (2006). Path-Dependent Human Identification Using a Pyroelectric Infrared Sensor and Fresnel Lens Arrays. Opt. Express.

[6] Johnson C. (2012). Mathematical Physics of BlackBody Radiation.

[7] Zungeru A.M., Mangwala M., Chuma J., Gaebolae B., Basutli B. (2018). Design and Simulation of an Automatic Room Heater Control System. Heliyon.

[8] Aaurav K., Ch andan V. Gray-Box Approach for Thermal Modelling of Buildings for Applications in District Heating and Cooling Networks. Proceedings of the 8th International Conference on Future Energy Systems.

[9] Purdon S., Jurdak B.K.R., Challen G. Model-free HVAC control using occupant feedback. Proceedings of the 38th Annual IEEE Conference on Local Computer Networks.

[10] Asgari H., Chen X.Q., Menhaj M.B., Sainudiin R. (2013). ANN-Based System Identification, Modelling and Control of Gas Turbines—A Review. Adv. Mater. Res..

[11] Wiener N. (1959). Non-Linear Problems in Random Theory. Physics Today.

[12] Schetzen M. (1980). The Volterra And Wiener Theories of Nonlinear Systems.

[13] Takagi T., Sugeno M. (1985). Fuzzy Identification of Systems and Its Application to Modeling and Control. IEEE Trans. SMC.

[14] Boussaada Z., Curea O., Remaci A., Camblong H., Mrabet Bellaaj N. (2018). A Nonlinear Autoregressive Exogenous (NARX) Neural Network Model for the Prediction of the Daily Direct Solar Radiation. Energies.

[15] Brus L. Nonlinear Identification of a Solar Heating System. Proceedings of the IEEE Conference on Control Applications (CCA 2005).

[16] Rabbani M.J., Hussain K., Khan A.U.R., Ali A. (2013). Model Identification and Validation for a Heating System Using Matlab System Identification Toolbox. IOP Conf. Ser. Mater. Sci. Eng..

[17] Lahane J.S., Meera A.K. (2014). System Identification and Controller Design for Boiler and Heat Exchanger Set-Up. Int. J. Adv. Res. Electr. Electron. Instrum. Eng..

[18] Knudsen M.D., Rasmus E., Hedegaard T.H. (2017). Pedersen, and Steffen Petersen. System Identification of Thermal Building Models for Demand Response—A Practical Approach. Energy Procedia.

[19] Building Controls Virtual Test Bed. https://simulationresearch.lbl.gov/projects/building-controls-virtual-test-bed.

[20] Open Modelica. https://www.openmodelica.org/.

[21] Van Overschee P., De Moor B. (1994). N4SID: Subspace Algorithms for the Identification of Combined Deterministic-Stochastic Systems. Automatica.

[22] Gao Y., Er M.J. (2005). NARMAX time series model prediction: Feed-forward and recurrent fuzzy neural network approaches. Fuzzy Sets Sytems.

[23] Johansen T.A., Foss A.B. (1993). Constructing NARMAX using ARMAX. Int. J. Control.

[24] Billings S.A. (2013). Nonlinear System Identification: NARMAX Methods in the Time, Frequency, and Spatio-Temporal Domains.

[25] Rumelhart D.E., Hinton G.E., Williams R.J. (1986). Learning Internal Representations by Error Propagation.

[26] Hastie T., Tibshirani R., Friedman J. (2009). The Elements of Statistical Learning: Data Mining, Inference, and Prediction.

[27] EnOcean—The World of Energy Harvesting Wireless Technology, January 2016. https://www.enocean.com/fileadmin/redaktion/pdf/white_paper/WhitePaper_Getting_Started_With_EnOcean_v1.0.pdf.

[28] Sadeghian Broujeny R., Madani K., Chebira A., Hurtard L. A multi-layer system for smart-buildings’ functional and energy-efficiency awareness: Implementation on a real five-floors building. Proceedings of the 2017 IEEE 8th International Conference on Awareness Science and Technology (iCAST).

[29] Sadeghian Broujeny R., Madani K., Chebira A., Hurtard L. A Machine-Learning Based Approach for Data-Driven Identification of Heating Dynamics of Buildings’ Living-Spaces. Proceedings of the 10th International Conference on Intelligent Data Acquisition and Advanced Computing Systems (IEEE/IDAACS 2019).

[30] WAGO Kontakttechnik GmbH and Co. KG, WAGO-I/O-SYSTEM 750 Manueal, 2016. http://www.safetycontrol.ind.br/imgs/downloads/manual-750-406-pdf-5b211d6fe9baa.pdf.

